# Post Surgical Non-tuberculous Mycobacterium: A Case Series

**DOI:** 10.7759/cureus.24701

**Published:** 2022-05-03

**Authors:** Ashok N Mhaske, Shubhangi Mhaske, Sanjay Harke, Arti Jain, Jaswant Patel, Sumedh Mhaske

**Affiliations:** 1 Department of Surgery, People's College of Medical Sciences and Research Centre, People's University, Bhopal, IND; 2 Department of Oral Pathology and Microbiology, People's College of Dental Sciences and Research Centre, People's University, Bhopal, IND; 3 Department of Biotechnology, Mahatma Gandhi Mission (MGM) Institute of Biosciences and Technology, Aurangabad, IND; 4 Department of Microbiology, People’s College of Medical Sciences and Research Centre, People’s University, Bhopal, IND; 5 Centre for Scientific Research and Development, People’s University, Bhopal, IND; 6 Department of Medicine, Government Medical College, Aurangabad, IND

**Keywords:** non tuberculous mycobacterium, post-surgical wounds, pcr, genotyping, antibiotic susceptibility

## Abstract

Background

There has been an increase in non-tuberculous mycobacteria (NTM) infection reports in humans. Surgeons are concerned about the link between them and surgical site infections. As a result, it has been challenging to determine just how common this illness is. A two-year study by the authors examines the occurrence of NTM infections after different surgical procedures.

Design and methods

Researchers at a tertiary care hospital in central India performed this prospective study over two years. NTM was found in six of the 25 instances of post-surgical wound infections. Ziehl-Neelsen staining, Auramine O-Rhodamine fluorescence staining, Multiplex Real-Time PCR, and Genotyping were used to identify the species.

Results

*M. fortuitum, M. chelonae* and *M.kansasii* were isolated from discharge in 2 cases each respectively. NTM isolates evaluated for antibiotic susceptibility pattern were all sensitive to Clarithromycin (100%). One case with prolonged healing had to be intervened with amikacin along with clarithromycin.

Conclusion

NTM is an infection of uncommon nature which can occur following surgical procedures. Identification of these organisms through sensitive techniques and appropriate therapeutic regimen formulation must be done to tackle this growing menace in health care setups.

## Introduction

Mycobacteria that do not cause tuberculosis (NTM) date back to the time of Robert Koch when he first discovered them [1]. These microorganisms are known to live in various environments, including water, animals, soil, and dairy products [2]. The *Mycobacterium chelonae*, *Mycobacterium abscessus*, *Mycobacterium fortuitum* and *Mycobacterium smegmatis* groups are most often linked with infections after NTM surgical intervention worldwide [3].

NTM is often linked to the presence of a foreign substance. According to conventional wisdom, infections are more likely when compromised skin's integrity. Antiseptic and disinfectant resistance is making these illnesses a growing threat. Surgical wound and skin biopsy, Ziehl-Neelsen staining, and mycobacterial cultures are not regularly performed; hence this burden is exacerbated [4].

These organisms can produce biofilm because of their hydrophobic nature. When these microorganisms shed, they produce biofilms that increase the likelihood of infection [5]. The infection from surgical wounds can be prevented by adequately sterilizing tools and endoscopes. The literature depicts the contamination of instruments and surgical site infections [6].

Globally, nosocomial infection outbreaks and cases have increased in recent years [6]. There have been reports of NTM in post-operative wounds in India [7-11]. Literature suggests that NTM is becoming more common, yet it may be difficult to diagnose. Long-term medication and lowered tolerance make it more challenging to find new treatment measures [12]. Few studies have been conducted in this region of the nation. Species and subvariants must be identified since treatment differs from species to species and must even be recognized from NTM environmental pollutants. Because of this, quick identification and treatment are necessary.

Accordingly, in Bhopal, an India-based tertiary care center was chosen to research the prevalence of NTM.

## Materials and methods

Study design

The Department of Surgery at a tertiary care hospital in Bhopal, Central India, undertook a prospective, long-term investigation. Patients included were those who presented with chronic discharging sinuses in operating wounds from September 2019 to September 2021.

Ethical consent

According to the Helsinki declaration, the research was conducted following the guidelines. The Peoples College of Medical Sciences and Research Center's Institutional Ethical Committee approved the study's protocols. (IEC No. PCMS/OD/2019/470)

Study population

Patients suspected of post-surgical infection consequent to various surgical procedures, some with an implant in situ and some without, were recruited for the study purpose.

Inclusion criteria

Delay in wound healing, breakdown of wounds, discharge or inflammation in the wound site, presence of nodule within the vicinity of wounds and those resistant to antibiotic intervention were included.

Exclusion criteria

Emergency surgical cases and wounds developed within seven days after surgery were excluded. Immuno-compromised individuals and participants not consenting to the study were not included.

Data collection

A case sheet elaborating on pre-operative and post-operative history and clinical examination was recorded for all cases.

Sample collection

A total of six swab discharge samples were collected from the deepest possible point of the wound. The swabs under strict aseptic measures were evaluated in our dedicated Biosafety Level II (BSLII) laboratory for mycobacterium evaluation.

Microbiological investigation

The samples were taken one by one as they were recruited. Gram stain smear was prepared from the first sample. Similarly, Ziehl Neelsen (ZN) and Auramine O - Rhodamine fluorescent staining (Acridine Orange) were done from the second and third samples. Genotype NTM-DR Version 1.0 to detect NTM used the fourth sample. Culture and Antimicrobial Susceptibility tests were processed from the fifth and sixth samples.

Sample processing

Using N-Acetyl-L-Cysteine - Sodium Hydroxide as a decontaminant, samples were centrifuged at 3000 rpm for 15-20 minutes before being suspended in 2 ml phosphate buffer for further analysis. The obtained supernatant and the decontaminated sample were then processed by real-time PCR test to demonstrate NTM at the infection site. Specimens obtained were subsequently cultured on Culture Media - 7H11, 7H9, and LJ (Lowenstein-Jensen) media to identify Rapidly Growing Mycobacteria (RGM) isolates.

Antibiotic susceptibility testing (AST)

AST was determined on Mueller-Hinton agar by the disc diffusion Kirby Bauer method for antibiotics discs of Clarithromycin, Erythromycin (15 ug), Amikacin (30 ug), Gentamicin (10 ug), Imipenem, Linezolid, Cefoxitin, Tetracycline (30 ug), Vancomycin (30 ug), Ofloxacin (5 ug), Ciprofloxacin (5 ug), cotrimoxazole (25 ug) and polymyxin B (300 ug).

Identification, typing, and characterization of isolates

NTM isolates were identified by line probe assay employing a multiplex real-time PCR system.

## Results

Twenty-five patients with post-surgical infections were included in this research, with an average age of 41.23 years. Patients that tested positive for NTM were on average 37.33 years old, with a ratio of four men to two women. Surgical interventions are enumerated in Table 1.

**Table 1:** Outcome summary of six NTM cases ZN - Ziehl-Neelsen; LJ - Löwenstein–Jensen; M - Mycobacterium; NTM - Non-tuberculous mycobacteria

Variables	Case 1	Case 2	Case 3	Case 4	Case 5	Case 6
Age (in years)	44	34	15	35	28	68
Surgical predisposition	Operation at the right gluteal region	Laparoscopic port site infection following cholecystectomy	Primary repair of the knee following a Road Traffic accident (Metal-on-metal implant)	Surgical intervention in the right leg (Metal-on-metal implant)	Surgery of the neck region	Shoulder implant surgery (Metal-on-metal implant)
Clinical presentation	Pus discharge	Discharge	Discharging sinus	Abscess with drainage	Sinus discharge	Sinus discharge present at two locations – elbow and shoulder
Gram staining	Negative	Negative	Negative	Negative	Negative	Negative
Z N staining	Negative	Positive	Positive	Positive	Positive	Positive
Auramine staining	Positive	Positive	Positive	Positive	Positive	Positive
Genotyping of species	M. kansasii	M. fortuitum	M. kansasii	M. chelonae	M. chelonae	M. fortuitum
LJ medium growth (in days)	9	6	9	6	9	8
Antibiotic intervention	Clarithromycin	Clarithromycin	Clarithromycin	Clarithromycin	Clarithromycin	Clarithromycin + Amikacin
Response Healed / Persisting	Healed in 150 days	Healed in 91 days	Healed in 164 days	Healed in 53 days	Healed in 38 days	Elbow location lesion healed in 18 days. Lesion at shoulder healed in 181 days

Clinical manifestations noted were pus discharge, abscesses, sinus tracts, or chronic discharge from the infection site. No other signs and symptoms such as fever and chills were found. Routine blood investigations were regular in all positive cases.

NTM was found in six of 25 cases accounting for an incidence of 24%. The time for the onset of infection after the surgical procedure was 19 days and for bacterial growth was 8.5 days. NTM isolates stained negative for Gram staining while positive in five cases for ZN and all for Auramine staining. All NTM cases were genotypically confirmed; the species isolated were *Mycobacterium fortuitum*, *M. chelonae*, and *M. kansasii*.

All isolates were sensitive to clarithromycin (CLR), while only one NTM confirmed case required Amikacin added along with CLR, which was coincidentally associated with diabetic comorbid conditions. No surgical treatment was given along with clarithromycin to facilitate the healing of the wounds. Clarithromycin was given for two months in these six cases.

The mean duration of follow-up of the patients after the initiation of clarithromycin therapy was a minimum of six months for all cases. The lesions did not recur in any of the patients after stopping clarithromycin. The mean healing of wounds was 11.283 days.

The patients treated are recovering at different rates, which is why species that have tested positive have hampered the healing process in various ways and to varying degrees.

## Discussion

Previous studies report an incidence of NTM ranging from 3.4% to 24.7% in India. An increase in reporting is due to better adaptation of histopathology, adherence to culture technique, and species diagnosis with DNA sequencing. Surgical predisposition varied amongst cases, with no tendency for either closed or open surgeries [13].

Culture sensitivity to ZN staining was detected to 83.33% of confirmed NTM infections. This was in non-concordance to the study of Ghosh et al. [14], who reported sensitivity in a lesser number of cases. This was attributed to inappropriate sampling technique, which inhibited the transfer of highly hydrophobic microbe to solid media or inadequate pretreatment before sample collection.

Generally, the ZN stain and culture are extensively used for diagnosing mycobacterial infections. AFB staining is a fast and inexpensive approach; however, it has poor sensitivity (22-78%), making it difficult to distinguish between NTM and *M. tuberculosis* [15]. Our study had 83% sensitivity to ZN staining. Advanced PCR techniques have 100% sensitivity and specificity for the DNA isolated from cultured specimens and can detect NTM faster than conventional PCR. The current study employed real-time PCR which targeted the 16S rRNA, rpoB, and hsp65 genes providing a success rate of 65%, 82%, and 87%, respectively [16]. Genotyping further enhanced the validity of our study as it could even identify *M. kansasii* in the 19 strip organism detection. NTM is categorized into four classes. Class 1 represents photochromogens that are slow-growing, such as *M. kansasii*. Microbes that cause cervical adenitis in children are Scotochromogens like *M. scrofulaceum* which belong to Class 2. All non-chromogens in Class 3 are included in this group, including *M. intracellulare*, *M. avium* (Cervical adenitis), and *M. xenopi* (chronic lung illness). Class 4 includes fast-growing microorganisms, including *M. fortuitum* and *M. chelonae*, which may cause chronic human abscesses [17]. The predominant isolate in our study was *M. fortuitum*, *M. chelonae*, and *M. kansasii*.

*M. kansasii* NTM isolates were found in our study, which is scarcely reported in published literature. First isolated in 1953, this yellow species colony carries a hereditary predisposition or shared environmental susceptibility. Conflicting reports of prevalence are found, suggesting significant regional variability. The enhanced sensitivity of Genotyping sequence (CM kitty) utilized in the present study played a pivotal role in identifying this rare colonizing organism [18]. Antibiotic susceptibility testing in the present study was by the disc diffusion method. RGM susceptibility pattern varies significantly with different species across the geographical location. It is recommended by the Infectious Diseases Society of America and the American Thoracic Society that all-important NTM isolates be tested for susceptibility before therapy can begin. Though micro broth dilution is the recommended choice by the Clinical and Laboratory Standards Institute (CLSI), the disc diffusion technique remains a reliable method [19-21].

Clarithromycin was effective against all NTM isolates. Amikacin was added along with clarithromycin in only one case of *M. fortuitum* infection. Hundred percent sensitivity of NTM to these drugs was also demonstrated in previous studies [22-24]. Our study did not require the usage of fluoroquinolones or multi-drug regimen comprising macrolide or aminoglycoside. Literature documents intralesional injections as well, the need for which did not arise in the current study [23]. All cases responded to Clarithromycin 500 mg BD. The only exception was the NTM infection in the shoulder implant surgery case, which took longer to heal and had lesions at two different sites, the elbow and shoulder location. The post-surgical wound of the elbow was sensitive to clarithromycin (CLR), while the shoulder lesion had to be treated with Amikacin four doses per week and took a long time to heal (181 days). In this context, emphasis is placed on reporting NTM infection and sensitivity patterns.

NTM infection increases due to inappropriate disinfection or sterilization of reusable medical equipment [25].

The multifactorial nature of infection cannot be overlooked. Though all surgical interventions followed similar infection control protocols during surgery and were exposed to the same environmental situation, the sporadicity of cases manifesting in only a few cases remains unclear. Our study addressed confounding issues by rigid inclusion of cases not to include immunosuppressed. The response of the individual human host, variation of NTM pathogenicity, and virulence are certain factors to be borne in mind.

In the context of the study, the limitations included the exclusion of comorbid condition patients, and the sample size was minimal, which can be dealt with just as a pilot trial. There is a significant limitation concerning the species tested that could be tested for more species.

## Conclusions

NTM infection is vastly implicated in surgical wound infections. Difficulty in diagnosing and prolonged morbidity caused by these microorganisms further add to the burden of the existing health care setup. The need of the hour is to increase awareness regarding the rising occurrence of NTM post-surgically, as it plays a significant role in the therapeutic regimen. Culture sensitivity and species identification are mandatory to provide optimal antimicrobial intervention. Surgical care for patients with recurring skin and soft-tissue infections necessitates understanding the risk.
